# Enterovirus-D68—Neglected Pathogen in Acute Respiratory Infections: Insights from Croatia

**DOI:** 10.3390/pathogens15010048

**Published:** 2026-01-01

**Authors:** Zeljka Hruskar, Lucija Skara Abramovic, Ivana Ferencak, Dragan Juric, Josipa Lozic, Anita Juric, Bojana Bocka, Marin Bajek, Mirela Josipovic, Viktor Bekic, Irena Tabain

**Affiliations:** 1 Microbiology Department, Croatian Institute of Public Health, 10000 Zagreb, Croatia; zeljka.hruskar@hzjz.hr (Z.H.); lucija.skara@hzjz.hr (L.S.A.); ivana.ferencak@hzjz.hr (I.F.); dragan.juric@hzjz.hr (D.J.); josipa.lozic@hzjz.hr (J.L.); anita.misic@hzjz.hr (A.J.); bojana.bocka@hzjz.hr (B.B.); viktor.bekic@gmail.com (V.B.); 2 Microbiology Department, Andrija Stampar Teaching Institute of Public Health, 10000 Zagreb, Croatia

**Keywords:** Enterovirus D68, respiratory infections, epidemiology

## Abstract

Background: Enterovirus-D68 (EV-D68) was long underreported, with only sporadic cases of respiratory disease worldwide until 2014, when numerous countries experienced significant outbreaks of EV-D68. In Croatia, sporadic detections have primarily resulted from an absence of systematic surveillance. Following the increased incidence of EV-D68 across Europe in 2022, we started to characterize EV-positive respiratory samples in Zagreb to confirm the presence of EV-D68 and identify circulating lineages through phylogenetic analysis. Methods: Respiratory samples from individuals with acute respiratory infection and additional clinical symptoms were tested at the Virology Laboratory of the Croatian Institute of Public Health, and EV-positive samples were further screened using the real-time RT-qPCR method for EV-D68. VP1 sequences were obtained by sequencing and subsequently genotyped. Results: Between March 2022 and December 2024, EV was detected in 2048 respiratory samples. Annual distributions of EV detections were 656 (10.0%) in 2022, 785 (8.1%) in 2023, and 607 (7.4%) in 2024. EV-D68 was identified in 13.1% of EV-positive samples in 2022, 1.4% in 2023, and 19.6% in 2024. The peaks in EV-D68 circulation were observed in July (*n* = 24) and September (*n* = 24) in 2022 and in September 2024 (*n* = 62). Phylogenetic analysis of EV-D68 VP1 sequences revealed the presence of two major clades, A2 and B3. The sequences from 2022 clustered exclusively within clade B3, while in 2024 A2 clade was newly introduced. Conclusions: We confirmed the presence of EV-D68 in Croatia with circulating lineages corresponding to those detected elsewhere in Europe. The absence of routine testing has likely led to an underestimation of EV-D68 prevalence. These findings underscore the urgent need for ongoing surveillance and genomic characterization to clarify EV-D68 epidemiology in Croatia.

## 1. Introduction

Enterovirus D68 (EV-D68), classified as a non-polio-EV within the human enterovirus species D of the *Picornaviridae* family, remains an underrecognized yet significant respiratory pathogen [1]. Although initially isolated in the United States in 1962 from children exhibiting acute respiratory symptoms [2], EV-D68 was long underreported, with only sporadic cases of respiratory disease reported, until a major epidemiological shift in 2014 [3,4,5,6]. That year, in the late summer, in the United States (US) and Canada, EV-D68 emerged and caused large outbreaks of severe respiratory illness accompanied by neurological disorders, particularly affecting children [7].

Since then, subsequent reports revealed outbreaks in Europe, Thailand, China, New Zealand, and various African countries, underscoring its global impact [8,9,10,11]. The increased use of molecular methods for identifying infectious agents has led to higher detection rates compared to traditional EV isolation in cell culture, which was previously the primary method for viral detection. Consequently, retrospective studies have revealed that EV-D68 has been circulating more frequently than previously recognized [12]. Meijer et al. [13] retrospectively tested specimens collected from acute respiratory infections (ARI) surveillance in the Netherlands from 1994 to 2010, and demonstrated almost annual circulation of the virus, challenging the previous perception of EV-D68 as a rare pathogen.

Beyond respiratory symptoms, EV-D68 has been associated with acute flaccid myelitis (AFM), a severe neurological condition, although a definitive causal relation between EV-D68 infection and AFM remains elusive [14]. Localized outbreaks of EV-D68 in Europe, including instances of severe respiratory disease and AFM, were reported in 2014 [15]. Despite these concerning developments, monitoring of EV-D68 remains inconsistent. In Croatia, EV surveillance has traditionally focused on stool specimens, with respiratory samples tested only occasionally using a generic enterovirus reverse-transcriptase real-time polymerase chain reaction (RT-qPCR) assay. Stool samples were subjected to virus isolation in cell culture, followed by typing through a virus neutralization test using monovalent or pooled antisera provided by the World Health Organization, or by commercial immunofluorescence assays [16].

Consequently, EV-D68 detection has been infrequent, reflecting a broader global gap in systematic surveillance and research.

The COVID-19 pandemic provided an opportunity for us to integrate the multiplex-PCR respiratory virus panel as a standard method for our routine laboratory analysis as part of respiratory non-sentinel surveillance. This integration enabled us to consistently identify EVs throughout the entire year.

In response to the observed increase in EV-D68 detections across Europe in 2022, this study aimed to investigate EV-positive respiratory specimens collected at the Croatian Institute of Public Health (CIPH) to evaluate the circulation of EV-D68 in Zagreb and the surrounding regions. The specific objectives were (i) to confirm the occurrence of EV-D68 among respiratory cases in Croatia and (ii) to determine the genetic lineages of circulating strains through phylogenetic analysis. The findings could highlight the value of sustained EV surveillance, systematic respiratory testing, and molecular characterization of EV-D68 to enhance understanding of its transmission patterns and public health impact.

## 2. Materials and Methods

### 2.1. Sample Collection

In this study, we conducted a retrospective targeted characterization of EV-D68 in EV-positive respiratory samples collected from patients with ARI or additional symptoms and tested at the CIPH. The analysis was laboratory-based and relied solely on routinely collected diagnostic specimens and data; therefore, additional clinical information was not available.

All EV-positive specimens obtained between March and December 2022 were retrospectively tested for EV-D68. Afterwards, all respiratory samples (nasopharyngeal swabs, nasopharyngeal aspirates, oropharyngeal swabs, tracheal aspirates, and bronchoalveolar lavage fluids) that tested positive for enteroviruses between January 2023 and December 2024 were routinely screened for EV-D68. Demographic and clinical data, including patient age, sex, primary diagnosis, sampling date, and specimen type, were also collected.

### 2.2. Detection of Enteroviruses

RNA was extracted from a 200 µL sample using the GeneRotex 96 Nucleic Acid Extractor (Tialong, Xi’an, China). The Multiplex–Tandem (MT) PCR test for identifying respiratory viruses was conducted with the commercial panel of assays Respiratory viruses (16-well; REF 20602; AUS Diagnostics, Mascot, Australia).

The respiratory multiplex panel included 12 pathogens (Enteroviruses, Parechovirus, Rhinoviruses, Human Bocavirus, Influenza A and B viruses, Human Parainfluenza 1–4, Respiratory Syncytial Virus, Human Metapneumovirus, Human Adenovirus, Human seasonal Coronaviruses, and SARS-CoV-2).

### 2.3. Detection of EV-D68 by Real-Time RT PCR

In-house, reverse transcriptase real-time PCR (RT-qPCR) was carried out using the Quant Studio 5 Real-Time PCR System (Thermo Fisher Scientific, Waltham, MA, USA) with the following thermal profile: 5 min reverse transcription at 50 °C, 20 s denaturation at 95 °C, followed by 45 cycles of 3 s denaturation at 95 °C and 30 s annealing at 60 °C. PCR was performed in a total reaction volume of 20 µL using Taq Man Fast Virus 1-Step Master Mix (Thermo Fisher Scientific, Waltham, MA, USA), containing 5 µL of extracted RNA, 600 nM of forward primer, 600 nM of reverse primer, 200 nM of probe 1, and 200 nM of probe 2 [15]. The primer and probe sequences for EV D68 detection are provided in Table 1.

### 2.4. Sequencing and Bioinformatic Analysis

Extracted RNA was reverse transcribed into cDNA using SuperScript IV Reverse Transcriptase (Invitrogen™, Waltham, MA, USA) and random hexamers (New England Biolabs, Ipswich, MA, USA). For amplification of entire capsid sequence, two sets of primers described by Majumdar et al. were used [17] PCR was carried out using Q5 High-Fidelity DNA Polymerase (New England Biolabs, Ipswich, MA, USA) under the following thermal cycling conditions: 98 °C for 30 s, 42 cycles of 98 °C for 10 s, 55 °C for 30 s, 72 °C for 2 min, followed by a final extension at 72 °C for 2 min. PCR products were purified with MagSi-NGSPREP Plus magnetic beads (Magtivio, Nuth, The Netherlands). Sequencing libraries were prepared using the Illumina DNA prep kit according to the manufacturer’s instructions and sequenced on Illumina MiniSeq and Illumina NextSeq550 sequencing systems (Illumina, San Diego, CA, USA).

Quality control (QC) of sequencing data was performed using a combination of bioinformatics tools. FastQC v0.12.1 was first used to assess the quality of the raw sequencing reads [18]. Subsequently, reads with a Phred quality score below 20 were trimmed using fastp v1.0.1 [19]. The quality of the trimmed reads was re-evaluated with FastQC to verify improvement. Genome coverage and average depth of sequencing relative to the reference genome were determined using SAMtools v1.12. A consensus sequence was generated using BCFtools v1.12 [20].

### 2.5. Phylogenetic Analysis

Randomly selected and sequenced EV-D68 samples (*n* = 12) were further analyzed and compared with representative sequences from other European countries. Clade classification (A2 and B3) was determined with the Enterovirus Genotyping Tool [21] and further confirmed by sequence alignment with reference genomes and comparison with published literature. All non-Croatian sequences were retrieved from the NCBI GenBank database. To ensure consistent annotation and facilitate comparative analysis, each retrieved sequence was manually labeled with its corresponding clade designation (A2 or B3), country of origin, and year of collection, based on available metadata.

All sequences were aligned based on the VP1 coding region, a highly variable and antigenically relevant segment, commonly used for molecular typing of EVs [4]. Multiple sequence alignment was performed using MAFFT v7.505 with default parameters [22]. Phylogenetic analysis was conducted using the Maximum Likelihood method in IQ-TREE v2.2.0 with automatic model selection [23]. Node support was evaluated using 1000 bootstrap replicates. EV-D70 was used as an outgroup to root the tree. Final tree visualization was performed using Interactive Tree Of Life (iTOL) v6 [24]. 

### 2.6. Statistical Analysis

Statistical analyses and data visualization were conducted using SPSS software, version 23 (SPSS Inc., Chicago, IL, USA) and Excel (MS Office 2013, Microsoft, Redmont, WA, USA). For categorical variables, descriptive statistics were presented as counts and percentages. Results were compared using the Chi-square test with calculated standardized residuals, Mann–Whitney U-Test, Shapiro–Wilk test and Kruskal–Wallis H test. All *p*-values were calculated, and a value of less than 0.05 was considered indicative of statistical significance.

## 3. Results

### 3.1. Annual and Monthly Fluctuations in EV and EV-D68 Infections

Between March 2022 and December 2024, EVs were detected in 2048 respiratory samples submitted for respiratory viruses panel testing. Annual distribution of EV and EV-D68 positive samples is presented in Table 2.

The annual distribution of EV-D68 detections among EV-positive samples differed significantly across the study period (Pearson’s chi-square test, χ^2^ = 126.946, df = 2, *p* < 0.001). The proportion of EV-D68 among EV-positive detections differed significantly between years, with the lowest positivity in 2023 (1.4%) and the highest in 2024 (19.6%), while positivity in 2022 was 13.11%.

**Figure 1:**
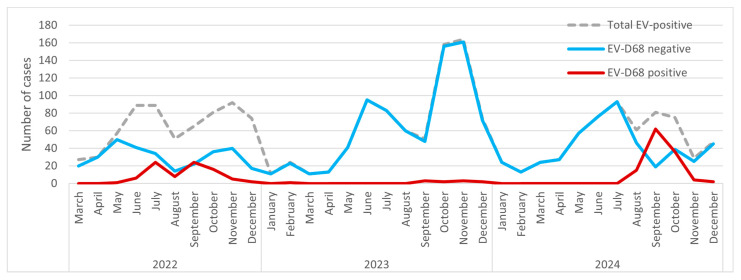
Monthly dynamics of EV-positive and EV-D68 positive cases.

The lowest EV activity was recorded in January and March 2023, with only 11 EV-positive detections (no EV-D68 cases). EV circulation showed a consistent seasonal pattern, with peaks usually occurring in June/July and October/November. The highest overall EV peak occurred in October and November 2023, with 158 and 164 EV-positive detections, respectively; however, only 2 and 3 of these were EV-D68-positive. By contrast, EV-D68 activity peaked in July and September 2022 (*n* = 24 each month) and in September 2024 (*n* = 62). Notably, EV-D68-positive detections exceeded EV-D68-negative detections in September 2022 (24/46; 52.2%) and September 2024 (62/81; 76.5%) (Figure 1).

### 3.2. Demographic Characteristics of EV and EV-D68–Positive Cases

Among EV-positive patients, the male-to-female ratios were 1.4:1 in 2022, 1.2:1 in 2023, and 1.7:1 in 2024. For EV-D68–positive cases, the corresponding ratios were 1.5:1, 1.8:1, and 1.4:1, respectively. Despite the observed differences, statistical analysis did not demonstrate a significant association between EV-D68 detection and patient sex (Fisher’s exact test, *p* = 0.75).

EV-D68-positive and EV-D68-negative patients were of similar age (Mann–Whitney U test, *p* = 0.5).

Chi-square tests showed that the distribution of age group differed significantly between EV-D68–positive and EV-D68–negative patients (χ^2^ = 13.573, df = 5, *p* = 0.019).

### 3.3. Primary Diagnosis of Enterovirus-Positive Patients

The most frequent primary diagnosis was febrile illness following acute upper respiratory tract infection in the EV-positive group across 2022, 2023, and 2024 (Table 3). For EV-D68 cases, febrile illness and upper respiratory tract infections were also predominant in 2022 and 2023. However, in 2024, lower respiratory tract infections became more prominent, representing 22.7% of EV-D68-positive cases.

Differences in primary diagnoses between patients with EV infection alone and those with detected non-EV coinfections were not analyzed.

Chi-square tests showed that the distribution of primary diagnoses did not differ significantly between EV-D68–positive and EV-D68–negative patients in 2022 and 2023 (2022: χ^2^ = 60.819, df = 6, *p* = 0.268, 2023: χ^2^ = 11.615, df = 6, *p* = 0.071). In contrast, the distribution differed significantly in 2024 (χ^2^ = 60.819, df = 6, *p* < 0.001).

### 3.4. Viral Coinfections

Among EV-positive patients, the majority were positive for EV alone (1510/2048; 73.7%). The annual proportions of samples without additional non-EV detections were 77.1% in 2022, 71.3% in 2023, and 72.1% in 2024 (Table 4). Further typing of EV-positive samples was not performed. Similarly, most EV-D68–positive cases showed no evidence of non-EV coinfection (181/216; 83.8%). A chi-square test showed that the frequency of non-EV coinfection among EV-D68–positive and EV-D68–negative patients differed significantly across study years (χ^2^ = 19.781, df = 2, *p* <0.001).

The most frequently observed coinfecting pathogens were adenoviruses, bocaviruses, respiratory syncytial virus (RSV), parainfluenza virus types 3 and 4, influenza A virus, and SARS-CoV-2. However, sporadic coinfections were also observed with human metapneumovirus, seasonal coronaviruses, rhinoviruses, and influenza B virus.

### 3.5. EV-D68 Sequences—Phylogenetic Analysis

**Figure 2:**
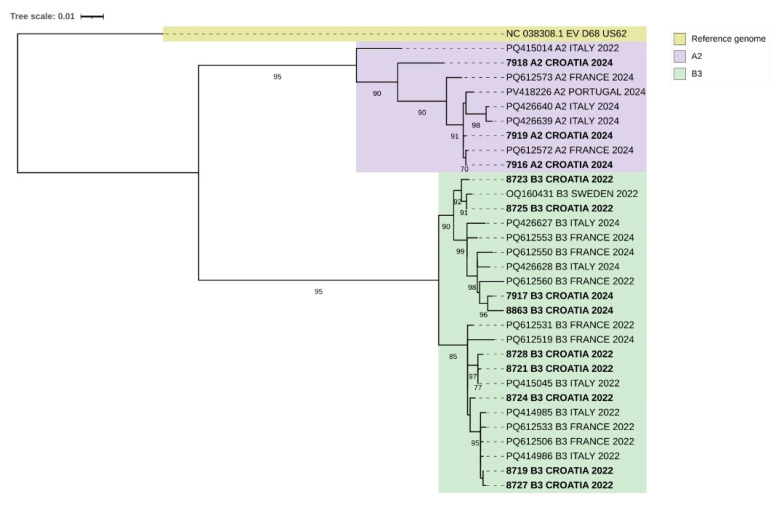
Maximum Likelihood phylogenetic tree of EV-D68 sequences based on the VP1 coding region. Randomly selected EV-D68 sequences from Croatia (highlighted in bold) were compared with representative European sequences retrieved from GenBank. The resulting topology clearly separates sequences into clades A2 (purple) and B3 (green), consistent with established EV-D68 genetic classifications. Visualization and annotation were performed using iTOL v6, with clade designations and reference genomes indicated by color coding. Numbers below the branches indicate bootstrap values.

Phylogenetic analysis of EV-D68 VP1 sequences from Croatian samples collected in 2022 and 2024 revealed the presence of two major clades, A2 and B3 (Figure 2). All Croatian sequences from 2022 clustered exclusively within clade B3, indicating the predominant circulation of this lineage during that period. Within clade B3, the sequences appeared to group into three distinct subclades, suggesting the co-circulation of closely related genetic variants.

In contrast, the 2024 Croatian sequences were distributed across both clades A2 and B3, suggesting either co-circulation of multiple lineages or the recent introduction of the A2 clade into the local population. Croatian isolates from 2024 grouped closely with sequences from other European countries, including France, Italy, Portugal, and Sweden, highlighting potential regional transmission events.

Bootstrap values supporting major nodes were above 70%, indicating strong confidence in the observed phylogenetic relationships. The tree topology also demonstrated clear separation between clades A2 and B3, with Croatian sequences interspersed within established European lineages.

## 4. Discussion

We presented findings on the detection of EV-D68 in Croatia from 2022 to 2024, following targeted analysis of EV-positive respiratory samples. By conducting additional characterization on all EV-positive samples, we were able to ascertain the presence of EV-D68 in respiratory samples.

Since its first isolation in 1962, the worldwide EV-D68 incidence was uncommon, and after 2014, outbreaks have become more frequently detected [2,5]. Due to the non-pharmaceutical measures implemented during the COVID-19 pandemic, the incidence of most respiratory viruses, including EV-D68, declined. However, its resurgence was detected in 2021 [25]. Our findings demonstrate the presence of EV-D68 in Croatia, corresponding to patterns of increased activity reported in the United States and other European countries during the study period [26]. Before this study, data on EV-D68 circulation in Croatia were unavailable. Moreover, because routine, systematic testing for EV-D68 was not performed, specific information on its circulation in 2021 is also lacking. This gap is notable, as 2021 saw an increased number of EV-positive samples in Croatia, alongside a rise in EV-D68 detections in EU countries, including neighboring countries, with a peak reported in October 2021 [27].

Enterovirus detection among respiratory samples tested with the standard virus panel varied seasonally (7.4–10.0%), with EV-D68 identified in 1.4–19.6% of EV-positive cases. Most of the EV-D68 cases were detected from June to October, which is consistent with previous data on EV-D68 seasonality in European countries, which usually peaks from September to October [28]. The very low detection of EV-D68 in 2023 is consistent with multicenter data indicating limited circulation in Europe compared with the major upsurges observed in 2018 and 2020–2021, with only sporadic reports of increased activity [29]. 

As of 2016, a comprehensive analysis revealed that systematic reporting and surveillance for EV-D68 had not been universally implemented across EU/EEA countries. Only 11 countries had established a surveillance system for EV-D68 infections, utilizing existing sentinel surveillance systems to type EV-positive respiratory samples or through acute flaccid paralysis (AFP) surveillance [30]. In our analysis, a respiratory surveillance system was utilized to include patients with ARI. However, in most EU/EEA countries, sentinel surveillance for respiratory pathogens is implemented, and this often includes non-hospitalized patients with either influenza-like illnesses (ILI) or ARI [31]. Therefore, in previously published studies, samples used for the detection of EV-D68 infection were collected via sentinel surveillance systems in Germany, the Netherlands, and Canada [10,32,33].

During the study period, only a small number of severe neurological cases associated with EV-D68 were identified, none of them was AFM (Table 3). By comparison, prior to this, just five cases of EV-D68–related acute flaccid paralysis had been reported in EU/EEA countries by the end of 2016 [30]. As highlighted by Harvala et al. [30] maintaining at least one dedicated system for EV-D68 surveillance is valuable for strengthening our understanding of the virus’s epidemiology.

Although more data are needed to show the epidemiological pattern of EV-D68 infections, previous investigations have suggested that EV-D68 infections might occur in a 2- to 3-year epidemic cycle [10,34]. Therefore, further monitoring and vigilant testing of EV-positive respiratory samples for EV-D68 should be continued.

Our findings demonstrate the presence of EV-D68 in Croatia, with reported cases exhibiting respiratory symptoms. These findings align with similar observations in both EU countries and the US, as highlighted in the studies by Benschop et al. [25] and Fall et al. [35]. Fall et al. emphasized the association of EV-D68 with respiratory symptoms in their research [35]. A prior study conducted in the EU further supports our findings by revealing that the majority of pediatric cases manifested respiratory symptoms, and notably, no cases of AFM were reported [25]. Despite EVs being recognized for their neurotropic nature, there were no neurological manifestations observed in cases associated with EV-D68. However, neurological symptoms usually occur with delays, as is shown in the findings from 2014 in the USA [36]. Although reporting of acute flaccid paralysis (AFP) is mandatory in Croatia, as in several EU countries [37], according to unpublished data from the CIPH, AFP surveillance relies primarily on ad hoc clinical reporting. Therefore, no AFM detection could be due to underreporting as well. As enteroviral infections may carry neurological implications, ongoing observation of EV-D68 could facilitate early recognition of any developing outbreaks and their potential clinical effects. Furthermore, it has been reported that viral evolution may influence the neurovirulence of enteroviruses [38]. Research by Smith Leser et al. found that VP1 is primarily associated with the neurological severity of the EV-D68 virus [39].

Phylogenetic analysis differentiates EV-D68 into four primary genotypes: A, B, C, and D (also referred to as A2). Since 2017, only two genotypes—B3 and A2/D2—have continued to circulate across Europe, Asia, and the United States [34,40,41,42] and there were no data on the circulating genotypes in Croatia. We have conducted genomic characterization of randomly selected samples. In our study, subclade B3 was dominant, especially during 2022, and then in 2024, B3 and A2 were identified, which is in line with previous similar findings from a study by Hirvonen et al. [29].

This study is subject to certain limitations. First, the absence of historical data on EV-D68 circulation in Croatia. Second, as this was a laboratory-based study, detailed clinical information was not available, and therefore, the clinical significance of EV-D68 detection could not be fully assessed. EV-D68 may also have circulated in patients with encephalitis or meningitis who did not present with acute respiratory symptoms, and most likely there were many asymptomatic cases. Furthermore, reliance on routinely collected samples limited the possibility of additional testing, so coinfections with non-viral pathogens could not be excluded. Finally, although only a limited number of EV-D68–positive samples were sequenced, phylogenetic analysis enabled us to establish links with strains detected in other European countries.

However, our findings highlight that EV-D68 has been underreported in Croatia, with its presence likely underestimated due to the lack of historical data and routine testing, and emphasize the urgent need for sustained systematic surveillance, including genomic characterization, and further research to fully understand the burden, epidemiology, and dynamics of EV-D68 in Croatia.

## 5. Conclusions

We confirmed the presence of EV-D68 in Croatia and, through phylogenetic analysis, identified lineages corresponding to those circulating in other European countries. Given the potential for severe clinical outcomes associated with EV-D68 infections, our results emphasize the need to strengthen such surveillance frameworks. Moreover, our findings illustrate that an integrated approach combining respiratory virus and EV surveillance is effective in detecting EV-D68 cases. This underscores the necessity for routine monitoring and systematic testing of respiratory samples for EVs, with EV-D68-specific analyses performed on positive specimens.

## Figures and Tables

**Table 1:** EV D68 primer and probe sequences.

Primer/Probe	Sequences (5′→3′)
EV-D68 Forward	TGTTCCCACGGTTGAAAACAA
EV-D68 Reverse	TGTCTAGCGTCTCATGGTTTTCAC
EV-D68 Probe 1	FAM-TCCGCTATAGTACTTCG-BHQ1
EV-D68 Probe 2	FAM-ACCGCTATAGTACTTCG-BHQ1

**Table 2:** Distribution of EV-D68 and Demographic Characteristics Among EV-Positive Samples, 2022–2024.

	Total Number of EV-Positive Samples	EV-D68 Negative	EV-D68 Positive	*p*
By year				
2022	656 (10.0%)	570 (86.9%)	86 (13.1%)	
2023	785 (8.06%)	774 (98.6%)	11 (1.4%)	<0.001
2024	607 (7.35%)	488 (80.4%)	119 (19.6)	
Demographic characteristics				
Female				0.75
2022	270	126 (78.8%)	34 (21.2%)	
2023	361	357 (98.9%)	4 (1.1%)	
2024	226	176 (77.9%)	50 (22.1)	
Male				
2022	386	178 (77.4%)	52 (22.6%)	
2023	424	417 (98.4%)	7 (1.6%)	
2024	381	312 (81.9%)	69 (18.1%)	
Median (months) IQR				
2022	28 (17–50)	26 (16–51.75)	32.5 (16–60.25)	0.50
2023	30 (18–54)	30 (18–54)	30 (18–150)	
2024	30 (18–54)	30 (18–54)	30 (18–54)	
Age groups (%)				
0–3 months	101 (4.9)	91 (4.9)	10 (4.6)	0.019
4–12 months	177 (8.7)	144 (7.9)	33 (15.3)	
13–24 months	528 (25.8)	478 (26.1)	50 (23.2)	
2–5 years	972 (47.4)	876 (47.8)	96 (44.4)	
6–15 years	213 (10.4)	192 (10.5)	21 (9.7)	
>16 years	58 (2.8)	52 (2.8)	6 (2.8)	

**Table 3:** Primary diagnosis of patients positive for enterovirus (EV) and EV-D68.

Primary Diagnosis	EV-Positive (%)	EV-D68 Positive (%)	*p*
2022			
Febrile illness	420 (64.0)	54 (62.8)	
Upper respiratory tract infection	120 (18.3)	17 (19.8)	
Lower respiratory tract infection	63 (9.6)	13 (15.1)	
Gastroenteritis	19 (2.9)	1 (1.2)	0.268
Enterovirus infection	18 (2.7)	1 (1.2)	
Rash	11 (1.7)	0 (0)	
Meningitis	5 (0.8)	0 (0)	
2023			
Febrile illness	441 (56.2)	6 (54.5)	
Upper respiratory tract infection	177 (22.5)	2 (18.2)	
Lower respiratory tract infection	22 (2.8)	2 (18.2)	
Gastroenteritis	29 (3.7)	0 (0)	0.071
Enterovirus infection	68 (8.7)	0 (0)	
Rash	35 (4.5)	1 (9.1)	
Meningitis	13 (1.7)	0 (0)	
2024			
Febrile illness	367 (60.5)	60 (50.4)	
Upper respiratory tract infection	111 (18.3)	25 (21.0)	
Lower respiratory tract infection	44 (7.2)	27 (22.7)	
Gastroenteritis	24 (4.0)	4 (3.4)	<0.001
Enterovirus infection	31 (5.1)	1 (0.8)	
Rash	24 (4.0)	1 (0.8)	
Meningitis	6 (1.0)	1 (0.8)	

**Table 4:** Virus (non-EV) coinfection in EV-positive and EV-D68-positive samples.

Year	EV- Positive Samples (%)	EV-D68 Positive Samples (%)	*p*
2022	150 (22.9)	20 (23.3)	
2023	225 (28.7)	4 (36.4)	<0.001
2024	163 (26.9)	11 (9.2)	

## Data Availability

The original data presented in the study are openly available from the European Nucleotide Archive at https://www.ebi.ac.uk/ena/browser/view/PRJEB105358 (accessed on 19 December 2025).
